# Toward Intelligent Roads: Uniting Sensing and Communication in Mobile Networks

**DOI:** 10.3390/s25030778

**Published:** 2025-01-28

**Authors:** Elisabetta Matricardi, Elia Favarelli, Lorenzo Pucci, Wen Xu, Enrico Paolini, Andrea Giorgetti

**Affiliations:** 1 Department of Electrical, Electronic, and Information Engineering “Guglielmo Marconi” (DEI), University of Bologna, 40126 Bologna, Italy; elia.favarelli@unibo.it (E.F.); lorenzo.pucci3@unibo.it (L.P.); e.paolini@unibo.it (E.P.); 2 WiLab, CNIT, 40133 Bologna, Italy; 3 Munich Research Center, Huawei Technologies Duesseldorf GmbH, 80992 Munich, Germany; wen.dr.xu@huawei.com

**Keywords:** 6G, cooperative sensing, joint sensing and communication, mobile radio networks, resource allocation, positioning and tracking

## Abstract

As 6G development progresses, joint sensing and communication (JSC) is emerging as a transformative technology, promising enhanced spectrum and energy efficiency alongside innovative services. This paper delves into underexplored facets of JSC, particularly its role in vehicular technology and transportation systems. It discusses data fusion techniques that enable cooperative sensing in networked environments and underscores the critical role of resource management in balancing sensing and communication. It suggests modeling extended targets, such as vehicles, within a computationally feasible framework. Moreover, it proposes a novel integration of AI-based target recognition, allowing target-specific tracking parameters and target-based sensing resource allocation. Importantly, a case study is presented to underscore the real-world applicability of these concepts in vehicular scenarios, demonstrating how networked devices can achieve high sensing and communication performance.

## 1. Introduction

The forthcoming advent of sixth generation (6G) cellular networks will mark not only a progression of current technologies but also a profound paradigm shift in communication principles. This endeavor aims to support ubiquitous sensing, seamless connectivity, intelligence, and the integration of innovative services with wireless devices.

Joint sensing and communication (JSC) holds great promise in increasing both spectrum and energy efficiency with acceptable hardware costs and complexity by sharing infrastructure, resources, and signals between communications and sensing [1,2]. It demonstrates the potential to foster communication-assisted sensing and sensing-assisted communication, thereby enhancing both functions to optimally support emerging applications within the 6G landscape. These applications encompass areas such as intelligent transportation systems, the protection of vulnerable road users (VRUs), traffic and environmental monitoring, and safety in urban scenarios, among others [3,4].

### 1.1. Literature Overview

While there has been extensive research dedicated to designing systems with high spectral efficiency and significantly improved performance from a communication perspective, there remain numerous unexplored aspects of JSC. For instance, while channel modeling in wireless communications has reached a mature stage, the same does not apply to sensing, which is often limited to simplified line-of-sight (LoS) models [5]. In [6,7], the authors propose leveraging 5G new radio (NR) vehicle-to-everything (V2X) sidelink communication for localization, exploiting the near-field propagation characteristics of signals generated at high carrier frequencies and large antenna arrays to enhance accuracy, robustness, and latency. In [8], the feasibility of JSC within the IEEE 802.11bd standard [9], an amendment to the IEEE 802.11 standard [10] designed to enhance V2X communications in challenging environments, was demonstrated. However, the impact of environmental factors, such as multipath effects, on sensing performance should also be evaluated. To make sensing an inherent network service, it is imperative to ensure its resolution meets the stringent requirements of emerging vehicular applications. Hence, a more descriptive model of the targets must be considered to represent real-world scenarios better when studying the requirements and performance of JSC systems [11,12]. In [13,14], a comprehensive method for jointly estimating channel and target parameters in multiple-input multiple-output (MIMO)-orthogonal frequency division multiplexing (OFDM) JSC systems was introduced, tackling a key challenge in the field. Although the method delivers precise estimates for channel parameters and point-like targets, its performance diminishes when handling a larger number of targets or extended target scenarios. Another crucial aspect is resource allocation, namely, assigning a portion of downlink or uplink resources to sensing tasks while ensuring that communication functions still satisfy quality-of-service (QoS) constraints. Although numerous studies have explored subcarrier and power allocation strategies that assign distinct subcarriers to sensing and communication tasks [15,16]—achieving significant gains in detection accuracy and communication capacity—it remains paramount to develop advanced resource allocation algorithms that consider contextual factors, such as specific QoS requirements and user equipment (UE) battery consumption [17]. This necessity further highlights the critical role of tracking in predicting the object positions, facilitating proactive and efficient network-wide resource allocation [18]. With the dense deployment of base stations (BSs) or transmission-reception points (TRPs) anticipated in future networks, there is immense potential to be unlocked through their cooperation when regarded as distributed sensors [19,20]. However, this collaborative sensing framework remains largely unexplored, and its potential to enhance the localization and tracking of vehicles, pedestrians, and other objects is still under-investigated.

### 1.2. Main Contributions

**Figure 1:**
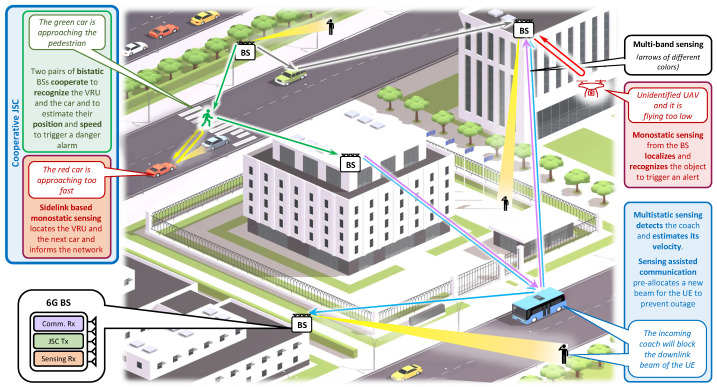
Urban scenario depicting monostatic, bistatic, and multistatic deployments where various targets, including pedestrians, cars, and UAVs, are detected, located, and tracked using JSC technology (figure background designed by macrovector/Freepik).

Motivated by the above-mentioned challenges, this article explores the integration of communication and sensing from a pragmatic perspective, with a focus on the role of JSC in real-world applications like vehicular technology. We outline the key challenges and benefits associated with different sensing configurations and present a case study that addresses some of these issues. Specifically, we introduce a data fusion technique that enables cooperative sensing among multiple monostatic JSC BSs within an MIMO-OFDM framework by exchanging soft maps with an fusion center (FC) at the network edge. The proposed system is evaluated in a challenging urban environment, employing a channel model that captures complex propagation effects, including multipath and multiple scattering points, to accurately represent extended targets such as vehicles. A key point of novelty of this work is the integration of extended target recognition using convolutional neural networks (CNNs), which is supported by a tracking algorithm that enhances the detection and estimation of both extended targets, like vehicles, and dim targets, such as pedestrians—an approach not previously explored in the literature for JSC MIMO-OFDM systems. Finally, the sensing/communication trade-offs are analyzed by varying the fraction of resources—power, frequency, time, and number of cooperating BSs—to provide insights into their impact on both the sensing and communication performance of the network. To narrow our focus within the vast JSC landscape, Figure 1 illustrates a typical urban environment where various JSC deployments can serve different purposes, such as detecting unauthorized unmanned aerial vehicles (UAVs), assisting in the preemptive detection of beam blockages, and enhancing road safety by detecting and locating vehicles and VRUs, including pedestrians and cyclists.

## 2. JSC Network Configurations

**Figure 2:**
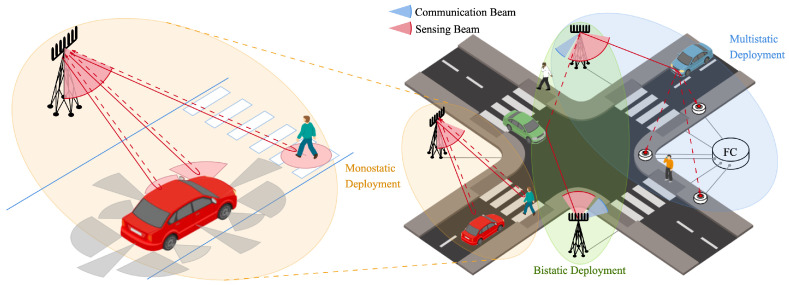
On the left, extended vehicle target model with distributed dispersers and pedestrian model with point-like reflector. The reflections depend on the relative orientation of the visibility cone with respect to the sensing beam. On the right, the three sensing configurations for a JSC system in an urban scenario.

There exist three primary setups for JSC systems, namely, monostatic, bistatic, and multistatic deployments (as depicted in Figure 2) that depend on the arrangement of transmitters and receivers. This section presents the distinct characteristics of these deployments and outlines their fundamental challenges.

### 2.1. Monostatic Deployment

The monostatic setup employs radio signals to survey the environment, capturing echoes from targets via a sensing receiver co-located with the transmitter. To illustrate, consider a BS that employs the echoes of its own data-carrying downlink communication signals to extract information about the surroundings. Addressing self interference (SI), effective beam management, and spurious targets is essential for safety-critical operations like collision avoidance and real-time object tracking when deploying such systems.

#### 2.1.1. Need of Full-Duplex Operations

Within a monostatic system, there is a need for full-duplex operations [1] with SI cancellation techniques to facilitate simultaneous transmission and reception, which occurs when the strong signal received directly from the transmitting antenna overshadows the weak signals reflected by the objects. Traditional sensing systems solutions, such as short transmitted pulses and idle periods for receiving echoes, are unsuitable for modern mobile communications systems and connected vehicles, requiring high spectral efficiency, low latency, and continuous environmental awareness.

#### 2.1.2. Single Beam Versus Multibeam

MIMO systems hold significant promise for achieving exceptionally high-capacity communication links by leveraging spatial multiplexing while simultaneously enabling real-time tracking of multiple objects, such as cars, cyclists, and pedestrians, on the sensing side. To utilize multiple antennas, the simplest approach involves phased arrays that perform sensing and communication in a time-division manner. Alternatively, substantial research has focused on developing beamforming strategies that facilitate the coexistence of communication and sensing beams [21]. This addresses the conflicting requirements of maintaining a stable communication link with the user equipment (UE) while allowing dynamic beam scanning on the sensing side to detect and track non-cooperative objects. Moreover, the alignment of transmitting and receiving antennas enables straightforward computation of range–angle maps through beam scanning.

#### 2.1.3. The Spurious Target Issue

In multibeam array systems, the emergence of ‘spurious targets’ is of significant concern. These can occur especially in the presence of strong beam sidelobes, e.g., during beam scanning. When sidelobes encounter strong reflections, they may cause the system to misinterpret the signal’s origin, resulting in ghost targets at unintended angles [22]. In dense traffic or city environments, ghost targets can cause the system to misperceive object locations, leading to safety risks. Advanced pruning algorithms are required to differentiate real objects (e.g., pedestrians or vehicles) from spurious targets to enhance the sensing system’s fidelity and mitigate the impact of unwanted artifacts caused by sidelobes.

### 2.2. Bistatic Deployment

Bistatic deployment defines a JSC system in which the transmitter and receiver are located at separate sites. Unlike monostatic configurations, bistatic setups are better suited for communication systems, eliminating the need for full-duplex architectures and SI cancellation techniques [23]. These configurations present a compelling opportunity to extend sensing areas while utilizing receivers that can be both simple and cost-effective. Consequently, bistatic JSC systems are particularly suited for vehicular scenarios. In vehicleto-vehicle (V2V) contexts, JSC facilitates information exchange among vehicles while simultaneously sensing nearby objects and vehicles. In V2X scenarios, sidelink sensing enables vehicles to detect and share information about their surroundings, including nearby vehicles [6,7], VRUs such as pedestrians, and other objects, as illustrated in Figure 1. These sensing applications are essential for collision avoidance, cooperative driving, and enhanced road safety.

#### 2.2.1. The Blind Zone Problem

In bistatic configurations, blind zones can occur in the area surrounding the baseline between the transmitter and receiver due to limited delay and angle resolution. This creates a minimum ellipse within which the transmitter-target-receiver path becomes indistinguishable from the direct path. In vehicular applications, these blind zones can jeopardize collision avoidance and object detection, particularly when vehicles are traveling at high speeds. The extent of the blind zone is influenced by the signal bandwidth and bistatic range resolution [24]. However, cooperation among sensors in the multistatic setup described below can help mitigate this issue.

#### 2.2.2. The Need for Synchronization

Given that the transmitter and receiver are not co-located, synchronization is essential. In these setups, precise synchronization is particularly critical for time-sensitive safety applications, such as cooperative driving. Potential solutions are being explored, including the implementation of a Cloud Radio Access Network (Cloud-RAN) architecture in mobile networks to ensure effective clock distribution. Additionally, the network infrastructure provides the receiver with information regarding the transmitted signal, e.g., pilots, necessary for signal processing tasks, such as matched or reciprocal filtering. Furthermore, in MIMO systems, achieving less stringent synchronization at the beam level is important to ensure that sensing beams at the transmitter and receiver are aligned, effectively targeting the same region of interest.

### 2.3. Multistatic Deployment

A multistatic configuration consists of spatially distributed transmitting and receiving devices, which can include both BSs and UEs, where each transmitter and receiver forms a bistatic pair. The observations made by receivers are shared with a FC, enhancing sensing performance compared to previous setups. Indeed, the radar cross-section (RCS) is typically highly dependent on the incidence and scattering angles and can exhibit low values or even notches in certain directions. Therefore, collecting echoes from the same object at different angles ensures a diversity gain. In addition to the synchronization challenges inherent in bistatic configurations, a well-designed spatial layout, effective data fusion methods, and strategic scheduling are essential in a distributed configuration. However, unlike bistatic deployments that are affected by the blind zone problem, the multistatic configuration allows the FC to leverage information from multiple bistatic pairs, effectively mitigating this issue. If a target is within the blind zone of one bistatic pair, it can still be detected and located by other pairs.

### 2.4. Information Fusion in Multisensor Systems

In multisensor systems, whether combining several monostatic BSs or using a multistatic setup, efficient data fusion is crucial and can be categorized into two approaches based on the information shared with the FC. In the first approach, individual sensors (e.g., a BSs or bistatic pair receivers) independently perform target detection and parameter estimation based on observations such as signal strength, time of arrival (ToA), angle of arrival (AoA), or time difference of arrival (TDoA) [25]. This method, known as hard fusion, minimizes network overhead but can result in lower detection and localization accuracy due to information loss and the lack of global data at individual sensors. The least square (LS) algorithm is commonly used to fuse individual estimations but requires at least three sensors for 2D target localization, increasing the risk of missed detections at one or more receivers, particularly when weak target echoes are involved.

In the second approach, each sensor processes the reflected target echoes and transmits soft information to the FC, which then fuses this soft information from all nodes to perform detection and estimation tasks [26]. Soft fusion generally outperforms hard fusion by avoiding the loss of weak targets caused by individual sensor preprocessing. However, this comes at the cost of increased processing and signaling overhead.

Ultimately, the choice of JSC deployment depends on application requirements such as sensing accuracy, range, and system complexity. Monostatic setups, despite the challenges of SI and full-duplex operation, are well suited for simple environments with co-located sensing, such as on-board vehicle systems. Bistatic configurations are ideal for V2V communication and sensing, while multisensor systems offer the greatest flexibility and reliability for applications demanding high precision, such as cooperative driving and collision avoidance. However, these systems rely on a network infrastructure, which, fortunately, is already in place.

## 3. Cooperative Data Fusion in JSC Network for Vehicular Scenarios

**Figure 3:**
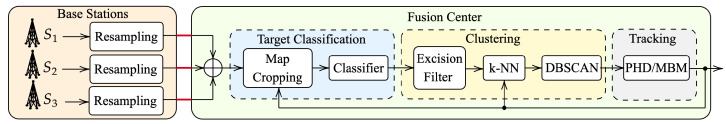
Block diagram of the sensing processing chain exploiting BS cooperation, target classification, and target-specific tracking. The BSs scan the environment, generating range–angle maps, and resample them according to a predefined grid. Resampled range–angle maps are then shared with the FC and fused in a single map. Target classification is performed at the FC through map cropping and classification (blue block). Then, clustering is performed to merge detections generated by the same target (yellow block). Finally, tracking algorithms perform target state estimation (gray block).

In this section, we present a comprehensive case study where multiple BSs cooperate to detect and locate vehicles and VRUs such as pedestrians and cyclists in an urban scenario. Through some numerical results, we aim to highlight key aspects of cooperative sensing in future mobile radio networks, including target and channel modeling, various fusion techniques, and resource allocation. Cooperative and distributed sensing technology aligns well with the anticipated network densification and massive deployments envisioned for 6G systems, making it a promising feature. The proposed cooperative data fusion strategy is summarized in the block diagram depicted in Figure 3.

### 3.1. Modeling Complex Targets: The Vehicle

The objects of interest for sensing, such as automobiles, bicycles, pedestrians, and drones, occupy a physical space in the real world. Whether an object is observed as a mere point or exhibits an extended spatial presence primarily depends on the sensor’s characteristics, particularly its resolution. When a sensor offers high resolution relative to the object’s size, it can yield multiple detections of the same target during each scanning cycle. These detections appear as spatially distributed measurements surrounding the object, whereas a lower-resolution sensor would lead to perceiving the object as a single scattering point.

#### 3.1.1. Vehicle Target Model

The modeling of an extended target such as a car has been and still is the subject of several studies, especially in the context of vehicular sensing systems. A relatively simple yet effective model approximating a vehicle with a rectangle featuring 12 point reflectors is provided in [27]. In particular, each scatterer is defined by its visibility function, which depends on the incident angle and RCS, and is positioned at specific locations, including the four corners, four wheelhouses, and four sides. The (dimensionless) visibility function takes the value 1.0 in the angular region, where the scatterer likely produces a high reflecting echo, gradually diminishing to zero in areas where the backscattered echo becomes feeble. The car sides are represented as point reflectors with narrow visibility functions and large RCS values. One example can be seen in Figure 2, where scatterers illuminated by the sensing beam reflect the signal if the visibility function is greater than 0. The RCS values of scatterers within an extended target (e.g., the 12 reflections of the car) or a point-like target are commonly described using statistical distributions from the Swerling family.

#### 3.1.2. Target Characterization via Measurements

Modeling complex objects as extended targets requires empirical validation through measurements. However, these models are typically designed for monostatic sensing and may not directly apply to multistatic scenarios. In monostatic systems, the reflected echo travels in the same direction as the incoming wavefront, whereas in multistatic systems, the AoA and angle of departure (AoD) values at the scatterer differ. Extended target models are especially critical in scenarios involving multiple cooperating BSs, where the multiview perspective of the target enables exploiting spatial diversity. For example, when observing a vehicle, a single BS may detect and estimate only a limited number of scattering points during each measurement, making it challenging to distinguish between extended and point-like targets. In contrast, multiple distributed sensors can reconstruct a more complete target representation. Despite our efforts to model cars in JSC systems, much of the existing literature does not account for the electromagnetic and geometric characteristics of extended targets due to a lack of suitable models. Thus, characterizing mobile targets is of paramount importance.

### 3.2. Multipath Effects on Sensing

The presence of multipath effects may significantly affect sensing, as it produces target smearing, i.e., even a point-like target is sensed as a superposition of several components with different delays, Doppler shifts, and AoAs values, which might blur the target. This aspect is particularly critical in vehicular scenarios, which are rich in multipath effects. To account for multiple paths, one can rely on deterministic channel models, e.g., ray tracing, which can accurately mimic the sensing channel but pose challenges in generating large-scale scenarios due to high computational requirements. Conversely, statistical channel models are much simpler but typically suffer a loss of spatial information, which is valuable for sensing.

Organizations like Third Generation Partnership Project (3GPP) strive to establish reference wireless channel models, aiding in assessing communication technologies [5]. Unfortunately, despite the considerable benefits and broad acceptance of such models, they do not fully capture the characteristics of the sensing channel, especially for monostatic setups [28]. In fact, while sensing in a bistatic configuration resembles a communication setup, in monostatic sensing, the transmitter and receiver are colocated, and this arrangement is not contemplated in current communication channel models.

It is important to note that the ETSI Industry Specification Group (ISG) on Integrated Sensing and Communications (ISAC), established in 2023, is dedicated to developing advanced channel and target models for various 6G use cases, aiming to address the limitations of existing communication-based channel models.

### 3.3. Monostatic Multisensor Data Fusion and Target Recognition

**Figure 4:**
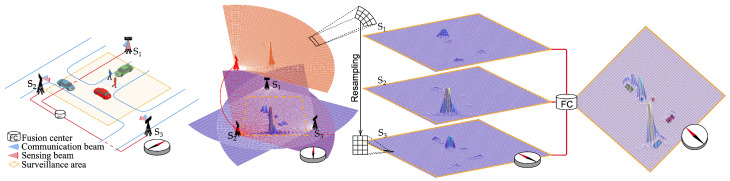
Fusion of range–angle maps from multiple BSs enables distributed and cooperative sensing, enhancing target detection and localization. Each BS detects nearby targets effectively, and fusion creates a global map of all targets for a comprehensive view.

An example of soft fusion in cooperative sensing systems, comprised of BSs acting as monostatic multibeam MIMO transceivers, is illustrated in Figure 4. In this example, targets are modeled as extended, and multipath effects are considered. The BSs provides range–angle maps shared with the FC. The considered system is a multibeam JSC scheme, with one beam directed toward the UE and another sequentially pointing in different directions to sense the environment. For each sensing direction *j*, starting from the frequency domain OFDM grid of received symbols, a periodogram can be computed to estimate the range and speed of the target by considering each grid element $g_{k}^{\left(m\right)}$, obtained at the *k*th subcarrier and *m*th time slot after reciprocal filtering, as follows [22]:

(1) $$P_{j}\left(q,p\right)={\left|\sum_{k=0}^{K_{p}-1}\left(\sum_{m=0}^{M_{p}-1}g_{k}^{\left(m\right)}e^{-j2\pi \frac{mp}{M_{p}}}\right)e^{j2\pi \frac{kq}{K_{p}}}\right|}^{2},$$

where $q=0,\cdots ,K_{p}-1$, and $p=0,\cdots ,M_{p}-1$. Here, $K_{p}$ and $M_{p}$ denote the next powers of two of *K* and *M*, respectively, which are the number of active subcarriers and OFDM symbols (or time slots). The term $g_{k}^{\left(m\right)}$ comprises two complex sinusoids per target, with frequencies related to $f_{D}$ (Doppler frequency) and τ (round-trip time). The periodogram defined in (1) generates a range–Doppler map, which serves as the basis for constructing range–angle maps. By assuming a receiving beamforming vector with a relatively narrow beamwidth, it is reasonable to consider that only one target is present in a given sensing direction. Consequently, the range profile r for the *j*th direction is extracted by isolating the column of the periodogram containing the peak value. Specifically, $r={\left[P_{j}\left(1\right),\cdots ,P_{j}\left(K_{p}\right)\right]}^{T}$, where $P_{j}\left(q\right)=P_{j}{\left(q,p\right)|}_{p=\hat{p}}$, and $\hat{p}$ represents the index of the column where the periodogram’s peak occurs. Repeating this process for all $N_{\mathrm{dir}}$ sensing directions produces a collection of range profiles, which are then organized into a matrix $R=[r^{\left(1\right)},\;r^{\left(2\right)},\cdots ,r^{\left(N_{\mathrm{dir}}\right)}]$. These maps, obtained at each BS, are then resampled to create a consistent grid. The resampled maps are then fused using an element-wise summation, followed by division by the total number of maps, to produce a unified map for target position inference. Here, soft fusion was employed instead of hard fusion to preserve information about the entire scene and avoid losing details about weaker targets, such as pedestrians. Notably, the fusion of range–angle maps reveals a dynamic interplay in target strengths. A signal that might be weak for one BS due to distance or low reflection amplitude can emerge as a strong detection for another. Conversely, seemingly strong targets, such as vehicles, may appear weak when located far from a receiver. The resulting composite image captures the intricate relationship among target strengths across the network, offering valuable insights into the diversity of the detections.

#### 3.3.1. Clustering for Extended Target Management

Clustering algorithms are commonly employed to manage multiple detections from extended targets, such as vehicles. In this work, we propose combining target state predictions (position and velocity) provided by tracking algorithms with a multistage clustering procedure. Initially, an excision filter can be applied to the soft maps to retain points with higher scores, which likely correspond to target reflections. In the next step, a k-nearest neighbors (k-NN) algorithm is used to cluster points that are likely associated with previously detected targets [29]. A gating strategy is exploited to associate new measurement detections with existing targets. Finally, the remaining points—those selected by the excision filter but discarded by the k-NN gate, i.e., those located too far from previously detected targets—are evaluated using the density-based spatial clustering of applications with noise (DBSCAN) algorithm [30]. DBSCAN defines the maximum distance for points to belong to the same cluster and the minimum number of points required to form a cluster. It is important to highlight that, in this context, DBSCAN is applied to cluster residual points that may represent potential new or emerging targets. Ultimately, the centroids of clusters formed by both k-NN and DBSCAN are stored, representing the target detections extracted from the soft map.

#### 3.3.2. Multiple Target Tracking

Tracking algorithms represent an essential tool in the context of JSC systems, as they enable continuous surveillance of moving objects and enhance target detection and localization accuracy owing to their ability to distinguish targets from clutter, especially in scenarios involving extended targets with multiple scatterers detected [31]. Furthermore, tracking can be vital in proactively allocating resources for dynamic target sensing, leveraging its capability to predict future target positions and behaviors.

Bayesian filtering theory is a prominent approach for implementing target tracking in JSC systems. This framework focuses on predicting the probability density function (p.d.f.) of a target’s state at a specific instant of time *t*, based on its state at the previous scan at time t−1. The predicted distribution is subsequently updated using measurements obtained at time *t*. To handle an unknown and variable number of targets over time, the probability hypothesis density (PHD) filter offers an efficient, yet effective, solution. In its Gaussian mixture (GM) implementation, this approach approximates the target intensity function as a GM with a predetermined number of components [32]. More specifically, an a posteriori target intensity function at time step t−1 is expressed as

(2) $$D_{t-1|t-1}\left(x\right)=\;\;\;\;\sum_{h=1}^{H_{t-1|t-1}}\;\;\;w_{t-1|t-1}^{\left(h\right)}N_{x}\left(\mu_{t-1|t-1}^{\left(h\right)},P_{t-1|t-1}^{\left(h\right)}\right),$$

where x is a target’s state; $H_{t-1|t-1}$ represents the number of Gaussian components in the prior intensity function; and $w_{t-1|t-1}^{\left(h\right)}$, $\mu_{t-1|t-1}^{\left(h\right)}$, and $P_{t-1|t-1}^{\left(h\right)}$ represent the importance, mean, and covariance of the *h*th component. The integral of the intensity function can be interpreted as an estimate of the number of targets present in the scenario. An alternative approach is the multi-Bernoulli mixture (MBM) filter, which leverages association probabilities between measurements and targets. The MBM distribution to represent the prior multiobject p.d.f. is given by

(3) $${\mathrm{MBM}}_{t-1|t-1}\left(x\right)=\sum_{g=1}^{G_{t-1|t-1}}w_{t-1|t-1}^{\left(g\right)}{\mathrm{MB}}_{t-1|t-1}^{\left(g\right)}\left(x\right),$$

where $G_{t-1|t-1}$ represents the number of multi-Bernoulli (MB) components or global hypotheses, and $w_{t-1|t-1}^{\left(g\right)}$ stands for the *g*th component importance. This filter demonstrates increased robustness in challenging scenarios, particularly in managing weak targets effectively [33].

#### 3.3.3. Target Recognition

While target recognition has been extensively researched for radar systems, a significant gap exists in the literature concerning vehicular scenarios, particularly in JSC systems that leverage communication-type waveforms. Target recognition is especially critical in scenarios involving dim targets, such as pedestrians. For instance, when a pedestrian is near a vehicle, the stronger reflections from the vehicle may overshadow those of the pedestrian, making it difficult for the system to maintain track of the pedestrian, even when its presence is known. In such cases, target recognition can fine-tune the tracking algorithm by extrapolating information about the obscured pedestrian from the available data. This is possible because each target produces a distinct reflection pattern based on its geometric shape and RCS, forming what can be described as its “reflection signature”. In this context, a CNN can be utilized to directly classify the target category based on the combined soft maps that contain this information [34,35]. Initially, image cropping for each target is performed, with each target framed within a square window of size $W_{\mathrm{size}}$ pixels, centered at the predicted target position at time *t*, which is obtained via tracking using information from the previous time step t−1. To generate a training dataset for the CNN, the actual positions and categories of the targets should be known. To improve the classifier’s performance and robustness against imperfect target state predictions, which may cause misalignment between the targets and reference frames, the true target position is perturbed during training. Gaussian noise, representing random displacement with a standard deviation $\sigma_{w}$ in both the *x* and *y* directions, is added to simulate this misalignment. By introducing variability into the training dataset, this approach enhances target classification accuracy during testing and reduces generalization errors, improving the system’s ability to perform well on unseen samples. Upon completion of the training phase, the CNN can perform real-time target classification in a variety of scenarios. This capability is highly beneficial for recognizing objects and making decisions based on the target category. For instance, the tracking measurement and prediction models can be adapted according to the specific target type. Numerical results demonstrating the advantages of target recognition for position estimation will be provided in the following section.

### 3.4. Resource Allocation Strategies

The sensing and communication capabilities of the BSs are ensured through shared resources between the two functionalities, including frequency division, time division, power division via a multibeam radiation pattern, and higher-level BSs selection. Since these two functionalities have contrasting requirements and performance metrics, ensuring proper resource allocation between them is critical. Assessing the sensing capability in a multitarget scenario, such as the one depicted in Figure 4, involves several metrics: missed detections, false alarms, and the localization error of detected targets. A good way to summarize performance into a single criterion is to use the optimal sub-pattern assignment (OSPA) metric, which incorporates the localization accuracy, detection rate, and false alarm rate into one measure. When no false alarms or missed detections are present (i.e., the number of detected targets equals the true number), the OSPA can be interpreted as the average root mean squared error (RMSE) of the position estimation [36]. Conversely, false alarms and missed detections introduce a penalty term, increasing the OSPA distance and reflecting degraded performance as errors accumulate.

For the communication capability, the aggregate capacity—defined in [18] as the downlink sum rate of each BS—can be considered. Another important aspect is the network overhead caused by inter-BS cooperation, which requires data exchange via backhaul to the FC; this is an issue that the research community has only recently begun investigating.

**Figure 5:**
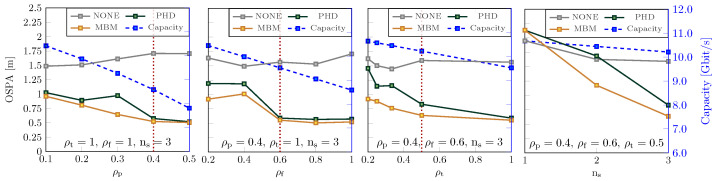
Sensing and communication capabilities versus the fraction of power $\rho_{p}$, the fraction of subcarriers $\rho_{f}$, the fraction of time slots $\rho_{t}$, and the number of sensors $n_{s}$. The PHD and MBM tracking algorithms (continuous lines) are compared to the case without tracking using OSPA (see the numerical scale on the left in meters). The average downlink aggregate capacity of the BSs is represented in blue (see the numerical scale on the right in bit/s).

Figure 5 shows the sensing/communication trade-off for a MIMO-OFDM system based on 5G NR numerology, where multiple monostatic BSs cooperate to detect and locate the vehicles and pedestrians in the urban scenario. Table 1 summarizes the main parameters of the waveform adopted and the vehicular scenario. Based on the different types of analysis of the JSC system, the following can be observed:

- *Impact of power division:* The fraction of power allocated for sensing when the system operates in a multibeam configuration is denoted as $\rho_{p}$. At low values of $\rho_{p}$, localization performance deteriorates due to the difficulty in detecting weak targets, such as pedestrians. Conversely, reducing $\rho_{p}$ enhances communication performance by increasing the available power. In this challenging scenario, the MBM filter outperforms the PHD filter.
- *Impact of frequency division:* Frequency division is achieved by allocating a fraction, $\rho_{f}$, of subcarriers for sensing. The choice of subcarrier allocation is crucial for sensing, as the range estimation resolution is highly dependent on signal bandwidth; generally, the larger the bandwidth, the higher the resolution. A small $\rho_{f}$ may result in target blurring and diminished localization capabilities. Conversely, reducing $\rho_{f}$ allocates more resources to communication, thereby increasing capacity.
- *Impact of time division:* Continuous scanning of the environment by the BSs may result in significant resource allocation for sensing, which is not always necessary. To conserve communication resources, the refresh rate of range–angle maps can be reduced by a factor D, leading to a fraction of time allocated for sensing, which is denoted as $\rho_{t}=1/D$. When $\rho_{t}$ is small, the sensing performance declines due to the tracking system’s limited ability to predict target motion accurately. Conversely, reducing $\rho_{t}$ increases communication capacity, as more time resources become available for this functionality. In this context, a key interesting feature of target classification is to adapt $\rho_{t}$ based on the target type; for instance, a pedestrian who moves at low speed can be tracked effectively with a $\rho_{t}=0.1$, while a car needs $\rho_{t}=0.5$, hence implicating more time resources.
- *Cooperation gain:* The number of cooperating BSs significantly affects sensing performance. In our case study, the OSPA decreased from over 2 m in the non-cooperative case (i.e., with $n_{s}=1$ BS) to 0.6 m when $n_{s}=3$ BSs were employed (see right-hand side plot in Figure 5). Clearly, allocating more BSs exclusively for communication purposes increases the overall network capacity.

Again with reference to Figure 5, it is worth noting that the vertical dotted lines indicate the values beyond which allocating additional resources no longer provides benefits for sensing and only results in a loss of resources for communication. Overall, the integration of the tracking algorithm, along with target recognition, led to significant performance improvements. For instance, in the scenario with $\rho_{p}=0.4$, $\rho_{t}=1$, $\rho_{f}=0.6$, and $n_{s}=3$, the OSPA metric improved markedly, reducing from 1.5 m without tracking to 0.5 m with tracking. This highlights the critical role of tracking in enhancing system performance, particularly in challenging scenarios involving closely spaced or extended targets.

**Figure 6:**
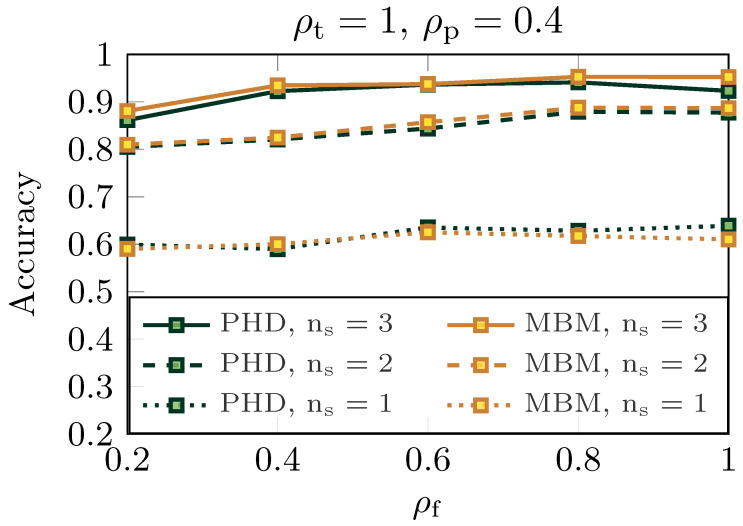
Target recognition accuracy versus the fraction of subcarriers $\rho_{f}$ fixing the fraction of power $\rho_{p}=0.4$ and the fraction of time slots $\rho_{t}=1$. Solid curves are generated considering $n_{s}=3$, dashed with $n_{s}=2$, and dotted using $n_{s}=1$. Green curves represent the PHD performance, while yellow stands for MBM accuracy.

Moreover, in Figure 6, the system’s target recognition capability was evaluated by varying the fraction of subcarriers and the number of monostatic BSs cooperating to classify the target. Classification performance is measured through accuracy, which is defined as the ratio of correctly classified targets by the CNN to the total number of objects detected by the tracking algorithms. The CNN was trained to recognize two types of targets: car or pedestrian. It can be observed that for both PHD and MBM, the CNN successfully identified the target category when the number of BSs was $n_{s}\geq 2$, with its accuracy exceeding 0.8. For example, with $n_{s}=3$ and $\rho_{f}\geq 0.4$, the accuracy rose above 0.9.

**Figure 7:**
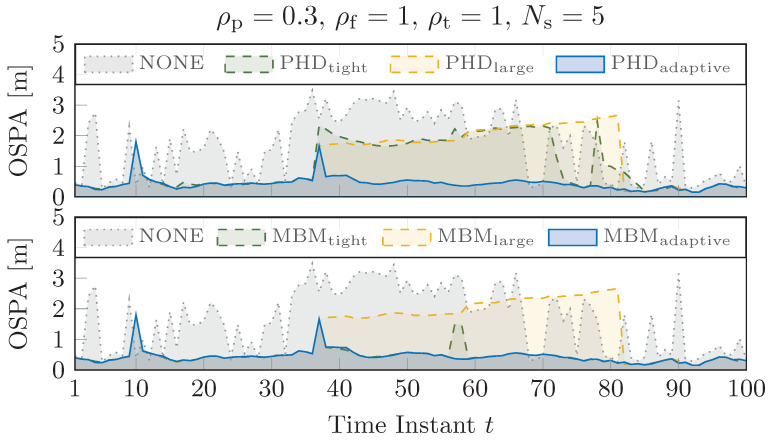
Localization performance (OSPA) over time for the PHD (**top**) and MBM (**bottom**). Target-type information is utilized by the clustering algorithms to adapt their measurement selection gates. The green dashed curves indicate a fixed tight gate, while the yellow dashed curves represent a fixed large gate. The blue solid curves correspond to adaptive gating based on target classification. Finally, the gray dashed curves represent the results obtained when tracking is not applied.

Finally, in Figure 7, the localization performance of cooperative sensing was evaluated over time by varying the clustering strategy and tracking algorithm. A significant performance improvement, indicated by stable OSPA values over time, was observed when the system’s target recognition capability was used to adapt the clustering gate for both tracking algorithms (blue curves compared to the green and yellow ones). This confirms the effectiveness of dynamically adjusting system parameters based on the target type.

## 4. Conclusions and Future Perspectives

We have provided a perspective on JSC, reviewing various setups—namely, monostatic, bistatic, multistatic, and multiple monostatic—while focusing on the dual functionality of communication and sensing, as well as the strengths and limitations of each configuration. We have emphasized the urgent need for a standardized JSC channel that incorporates multipath components and accommodates extended targets, particularly in complex and highly dynamic scenarios such as vehicular environments. Target classification has been addressed as a key feature to enable target-specific tracking and optimize resource allocation parameters. Additionally, we explored the intricate domain of resource management, considering the allocation of power, frequency, time, and the number of sensors. Finally, to demonstrate the benefits of synergy between resource allocation strategies, cooperative sensing, and target recognition, we presented numerical results in a realistic vehicular case study. The numerical results demonstrate that cooperation among three BSs enables the localization of both extended and point-like targets, with an error of less than 80cm, while maintaining a downlink capacity exceeding 10Gbit/s for a UE experiencing an signal-to-noise ratio (SNR) of 8dB when considering $\rho_{p}=0.4$, $\rho_{f}=0.6$, and $\rho_{t}=0.5$. While a large body of literature overlooks the role of tracking in JSC systems, our numerical results reveal significant improvements when tracking is incorporated, highlighting its critical contribution to the overall system performance. Additionally, the integration of the CNN for target recognition demonstrates its effectiveness in identifying target categories as either car or pedestrian, with its accuracy exceeding 0.8 for two cooperating BSs and surpassing 0.9 when three BSs cooperate and $\rho_{f}\geq 0.4$.

While this work addressed key challenges in cooperative sensing and data fusion for JSC systems, several promising avenues remain for future exploration. The data fusion technique proposed in this study, which enables cooperative sensing among monostatic JSC BSs via soft map exchange at a fusion center, could be further optimized through advanced beamforming strategies [37]. Specifically, integrating multicell coordination and distributed MIMO radar functionalities could balance sensing and communication objectives, enhancing waveform diversity and improving resolution in complex urban scenarios like the one evaluated in this work. Additionally, the use of reconfigurable intelligent surfaces (RISs) offers a complementary approach to overcome the propagation challenges highlighted in this study, such as multipath and occlusions [38]. By strategically deploying RISs, it would be possible to enhance the detection and tracking of shadowed or dim targets, including pedestrians, while simultaneously improving localization precision for extended targets like vehicles. Furthermore, the analysis of resource trade-offs in this study highlights the importance of exploring distributed sensing in cell-free massive MIMO systems. Deploying dense antenna arrays across a wide area could improve spatial resolution and enable fine-grained detection, especially for weak or extended targets. Scaling antenna deployments dynamically to match specific sensing needs could make such systems highly adaptable to a variety of applications, from traffic monitoring to cooperative autonomous driving.

## Figures and Tables

**Table 1:** JSC system and scenario parameters.

$f_{c}$ [GHz]	28	Beamwidth [deg]	2.4
*B* [MHz]	400	Number of BS $n_{s}$	3
Active subcarriers *K*	3168	EIRP [dBm]	30
Δf=B/K [kHz]	120	Surveillance area [m^2^]	50×50
OFDM symbols direc. $M_{s}$	112	Number of pedestrians	0 to 2
Number of antennas	50	Number of cars	2 to 3

## Data Availability

The code used in this study is proprietary and cannot be shared due to confidentiality restrictions. For inquiries, please contact the corresponding authors (elisabett.matricard3@unibo.it, andrea.giorgetti@unibo.it).
